# Case Report: Bilateral syringocele in an adolescent—area of focus

**DOI:** 10.3389/fped.2023.1239615

**Published:** 2023-11-24

**Authors:** I. B. Osipov, D. A. Lebedev, A. A. Uzintseva, N. A. Sidorova

**Affiliations:** ^1^ Department of Urology, Saint Petersburg State Pediatric Medical University, Saint Petersburg, Russia; ^2^ Department of Pathological Anatomy, Saint Petersburg State Pediatric Medical University, Saint Petersburg, Russia

**Keywords:** bilateral Cowper’s syringocele, bulbourethral glands—abnormalities, Cowper’s glands, endoscopic treatment, congenital urethral anomalies

## Abstract

**Rationale:**

Cystic dilatation of the bulbourethral gland duct (Cowper's syringocele, CS) is a rare urethral pathology. No more than 150 cases of CS have been reported in the literature, of which the vast majority are children with a unilateral location. Bilateral CS has been reported in eight cases; however, detailed anatomy and clinical manifestations have not been reported. In this study, we report a case of bilateral CS with cavity junctions through the medial septum and its successful minimally invasive treatment in an adolescent.

**Case presentation:**

A 16-year-old adolescent patient complained of painful urination and post-micturition urinary dribbling. Magnetic resonance imaging (MRI), urethrography, and ureteroscopy data enabled the establishment of the diagnosis and determination of the type of CS. The surgical treatment that was performed was endoscopic marsupialization of both CS chambers. At follow-up examination after 7 months, the complaints had ceased, and there was complete normalization of urination. The maximum flow rate during uroflowmetry was 35 ml per second, on voiding cystourethrography, the residual cavities were not contrasted, and there was no residual urine or bacteriuria.

**Conclusion:**

MRI and retrograde urethrography made it possible to visualize changes in the bulbous part of the urethra, and ureteroscopy was the leading imaging modality. These studies was applied precisely because of the suspicion of the presence of a cavity communicating with the urethra. Minimally invasive surgical treatment for double-chamber CS was successful with no resulting complications.

## Introduction

The bulbourethral glands were first mentioned by J. Mery in 1684 and subsequently described in detail by W. Cowper in 1699, after which they became known as the Cowper's glands (1). Secretion and emission from the Cowper's glands in humans is carried out through the cooperation of striated and smooth muscles (2). Cystic dilatation of these ducts is uncommon and is called “Cowper's syringocele” (CS) (3). The incidence of CS remains unclear. In 1849, Gubler described the first congenital case, in 1883, Englisch described the first perineal cyst, and in 1886, Elbogen identified an overall incidence rate of 2.3% from autopsy studies, with five of his 16 cases being almost certainly congenital (4). Dilation of the ducts of the Cowper's glands was first described in detail by P. S. Moskowitz. According to the author, fewer than 40 cases of CS had been reported in the literature by 1976 (5). Wagemans (6) performed urethroscopy on 4,000 patients and identified syringocele in 3% of them. Maizels et al. identified four types of CS based on radiological and endoscopic findings in eight patients: the author identified four types of cystic changes in the ducts of the bulbourethral glands: type A, simple syringocele—Cowper's duct—is visualized by simple reflux into a minimally dilated duct. Type B, imperforate syringocele—orifice draining Cowper's duct—is not apparent and is associated with dilatation of the distal duct. The ectatic duct intrudes into the bulbar urethra. Type C, perforate syringocele—orifice draining Cowper's duct—is patulous and allows free reflux into the duct. The ectatic duct appears as a diverticulum of the bulbar urethra. Type D, ruptured syringocele—bulbar portion of Cowper's duct—is dilated and loses its communication with the main duct. The rupture of this dilated duct leaves membranes as remnants of its wall (7). Bevers et al., in order to simplify the classification of Maizels et al., use only two types of CS: open and closed (8). Currently, open and closed forms of CS are more often distinguished. One article was found in the scientific literature mentioning double-side CS in eight patients, with no detailed description of the cases (6), and only once a description of the combination of open and closed forms in a patient with symptoms of postvoid incontinence, weak flow, and frequency was given (8). Javed et al. stated that the disorder may be inherited or acquired through trauma, urethral catheterization, or inflammation; however, the pathogenesis of the pathology remains unknown (9). Patients are treated only in the presence of symptomatic CS (painful urination, post-micturition dribbling, urethrorrhagia, paresthesia in the penis, lower abdomen, inner thighs, and infection), which is confirmed by radiological and endoscopic examinations (obstructive or perforated CS). Diagnosis is made using urethrocystoscopy or a retrograde urethrogram (8). Several methods of surgical correction of the defect have been studied in the literature, and laser marsupialization of the anterior wall of a cystic dilated duct is more often used. Usually, removal or dissection of the cyst walls is performed, in some cases, after prior urine diversion in cases of significant infravesical obstruction (3, 10, 11).

## Clinical case presentation

**Figure 1:**
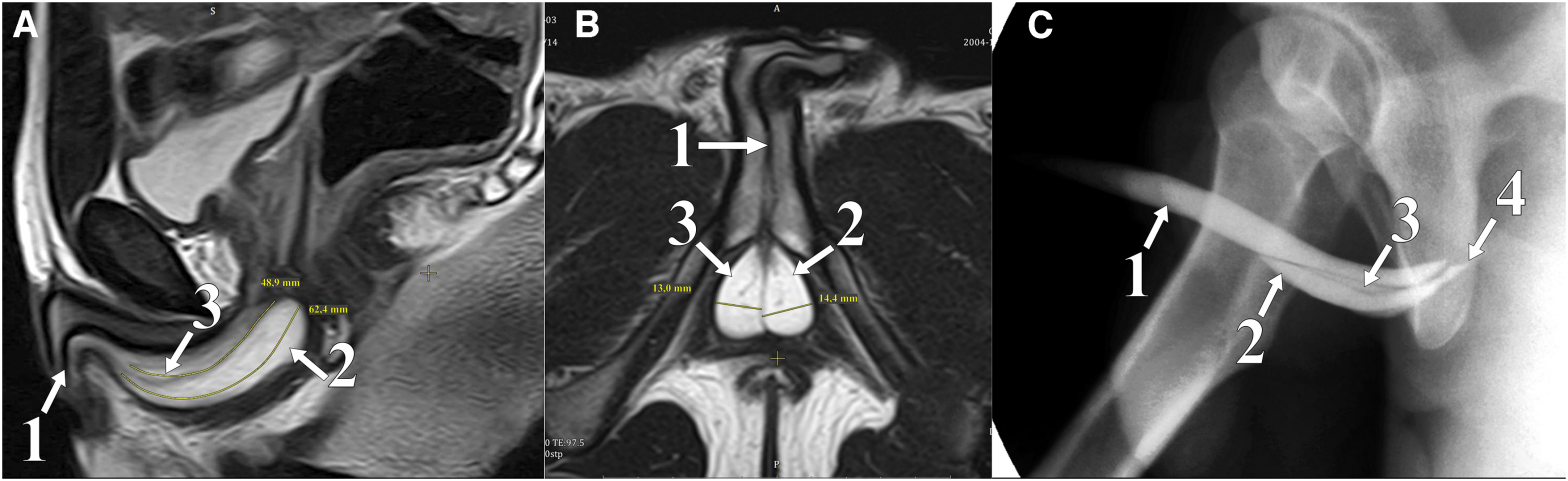
Lateral (**A**) and sagittal (**B**) plane MRI: T2 WI mode; soft tissue in the pelvis. Retrograde urethrogram (**C**): two paraurethral cavities in the bulbar area contrasted. (1) Urethra, (2) left chamber CS, (3) right chamber CS, and (4) Cowper's duct.

**Figure 2:**
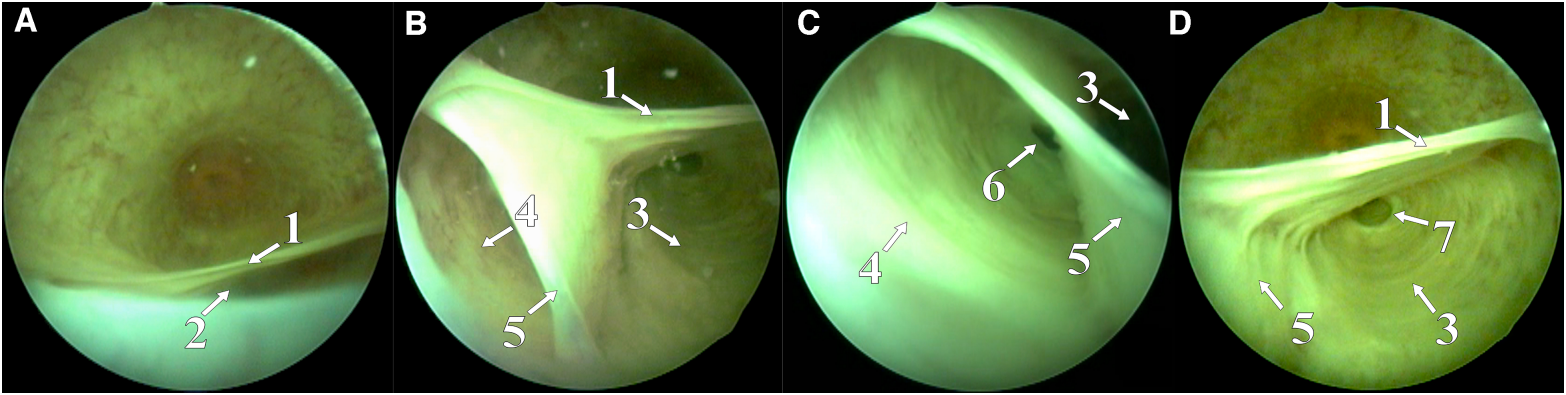
Hole in the left CS chamber (**A**), hole in the inter-chamber septum, through which the right CS was opened (**B**), and right (**C**) and left (**D**) CS chamber with gaping ducts of bulbourethral glands. (1) CS roof, (2) left CS hole, opening into the urethral lumen, (3) left CS chamber, (4) right CS chamber, (5) septum between the right and the left CSs, (6) duct of the right Cowper's gland, and (7) duct of the left Cowper's gland.

**Figure 3:**
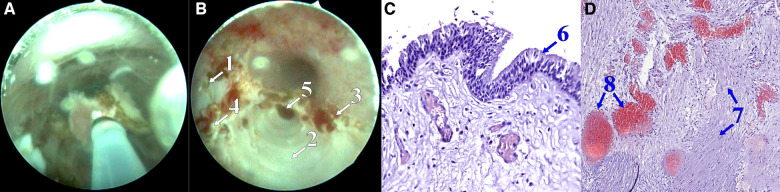
Removal of the septum between the cavities (**A**), postoperative view (**B**); microscope slide: hematoxylin-eosin staining, 20×, lined with prismatic epithelium (**C**); microscope slide: hematoxylin-eosin staining, 9.5×, the wall of the cyst is formed by fibrous tissue, with a large cistern of blood vessels of varying caliber and blood filling (**D**). (1) Bottom of the right CS chamber, (2) bottom of the left CS chamber, (3) edge of anterior wall resection, (4) edge of septal resection, (5) duct of the left Cowper's gland, (6) prismatic epithelium, (7) fibrous tissue, and (8) blood vessels.

**Figure 4:**
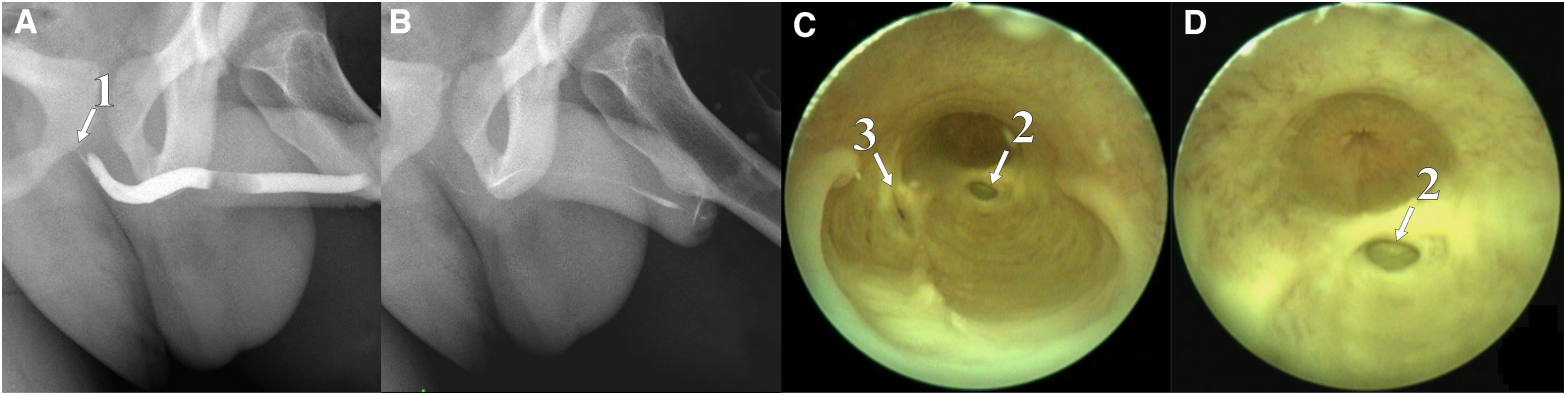
Retrograde urethrogram with tight urethral filling (**A**), contrast reflux into the bulbourethral duct is contrasted; after emptying, the urethra contains a small amount of remaining contrast (**B**); urethroscopy: back of CS chambers connected to the urethral lumen (**C**) and gaping of the left bulbourethral duct is visible (**D**). (1) Contrasted duct of the Cowper's gland, (2) duct of the left Cowper's gland, and (3) duct of the right Cowper's gland.

In November 2021, a 16-year-old boy was admitted to the urology department of Saint Petersburg State Pediatric Medical University. The patient demonstrated a clinical picture of painful urinary syndrome for the past 2 years, with post-micturition urinary dribbling of up to 10 ml for 3−4 min after each urination, without urinary tract infection (UTI) manifestations. Anamnesis: 2 years ago, hard seating on the chair, there was a short-term moderate urethrorrhagia and a severe urethral pain, followed by the appearance of post-micturition urinary dribbling, not accompanied by urinary urgency and frequency, nocturia, and persistent or intermittent pelvic pain in the absence of proven infection (12). There were no catheterization, surgical, or instrumental interventions on the urethra or bladder prior to the complaints. A radiological examination was carried out in the patient's region of residence. A magnetic resonance imaging (MRI) revealed two paraurethral cavities in the bulbous urethra: 62.4 mm × 14.4 mm on the left and 48.9 mm × 13.0 mm on the right. In retrograde urethrography, the cavities were contrasted from the urethral lumen, with the contrast agent thrown into the duct of the bulbourethral gland (Figure 1). There was no specific treatment for urinary dribbling, and therefore, admission to another hospital was recommended. When the patient was examined in our department, uroflowmetry was performed, which revealed that the urine flow rate was up to 38 ml per second, the median flow rate was 10.3 ml per second, and the bell-shaped flow rate showed no rapid amplitude changes, which was in the 25%–75% percentile range. There was no residual urine. Urethroscopy revealed an opening at 6 o'clock on the conditional clock leading into the left CS, with the right chamber opening into it through the common medial wall, and an examination of the chambers proximally revealed dilated ducts of the right and left bulbourethral glands (Figure 2). The CS on the left was larger than that on the right, which was preliminarily confirmed by an MRI. The double-chambered cyst was a urine reservoir, defining the clinical symptomatology and post-micturition urinary dribbling. Endoscopic marsupialization of the double-chamber CS and removal of the common medial wall, up to the hanging urethra, was performed. A 14 Fr rigid ureterocystoscope, with a 4 Fr light guide, 30 W YAG-Ni laser, and fibrotome mode, was used. As a result, a wide connection to the lumen of the urethra was achieved (Figure 3). A 14 Fr Foley catheter was placed after the operation. The duration of the postoperative stay, antibiotic prophylaxis, and catheterization was 7 days. An examination of the dissected CS walls showed that they contained edematous fibrous tissue, and the lining was composed of prismatic epithelium (Figure 3). The postoperative period was without complications. At follow-up examination after 7 months, positive dynamics were observed, and the patient's complaints of urinary incontinence and painful urination had completely ceased. The urine culture was sterile. No pathology was observed in the retrograde and voiding urethrography in the lateral view. After emptying, the urethra contained no remaining contrast. Upon control urethroscopy using a 14 Fr rigid urethrocystoscope, the lower parts of the chambers were visible in the CS resection area, with no formation of scar narrowing. The left bulbourethral duct retained a partial gaping (Figure 4). On uroflowmetry, the maximum urine flow was 35 ml/s, the median flow rate was 23.1 ml/s, and the voided percentage was 99. The volume voiding rate curve was bell-shaped, in the 25%–75% percentile (13). There was no significant residual urine. What makes this clinical case unique is precisely the extremely rare bilateral pathology of the excretory ducts of the Cowper's glands, with the formation of two intraurethral cavities of considerable size, which determine the clinical manifestations in the form of secondary post-micturition urine leakage.

## Discussion and conclusion

We treated the case described in the adolescent as bilateral perforated CS (type C according to Maizels et al. and open form according to Bevers et al.) (7, 8), with the chambers connected through the medial septum. The most likely cause of the pathology is genetic since inflammation and trauma of the urethra were not mentioned in the patient's history. No clinical description of this form of syringocele could be found in the available literature. A bilateral syringocele in eight children was described by Wagemans et al. without any clinical details (6). In this pathology, the pathognomonic symptom was post-micturition urinary dribbling (post-micturition dribble: this term is used when the child describes involuntary leakage of urine immediately after voiding has finished according to the ICCS) (14), following an episode of urethrorrhagia and urethral pain syndrome. Post-micturition urinary dribbling in patients with a perforated syringocele has been well described (9, 15). Differential diagnosis has been made with a prostatic utricle cyst and urethral diverticulum (16, 17). In our case, radiological and endoscopic examinations enabled accurate diagnosis and minimally invasive surgical treatment and to stop the leakage of urine in the patient. Diagnostic efficiency with the use of MRI, urethrography, and urethroscopy has been reported by many authors (8, 11). Treatment with the YAG-Ni laser has also been described in the literature (7), with endoscopic ablation and cyst wall marsupialization being the preferred surgical treatment options. In our patient, despite the significant amount of scar tissue in the removed CS fragments, postoperative healing was uneventful, and there was no formation of urethral scarring after surgery, which was confirmed by uroflowmetry data (the median urine flow rate almost doubled), the absence of residual urine, and the presence of a lumen of the urethra without narrowing by control urethroscopy.

In the case of post-vomiting urine dribbling, the patient is advised to undergo retrograde urethrography and urethroscopy to confirm the diagnosis of CS. Long-term observation after endourethral intervention is not required.

The presented case of a teenager with bilateral syringocele is important due to its extreme rarity and will, therefore, be of interest to pediatric urologists.

## Data Availability

The original contributions presented in the study are included in the article/**Supplementary Material**. Further inquiries can be directed to the corresponding author.

## Supplementary material

Supplementary File S1 Video presentation of this clinical case.

Supplementary File S2 CARE checklist.pdf (CARE checklist for this article).
